# Psoriatic Arthritis: Therapeutic Advances and Novel Treatment Strategies—A Scoping Review

**DOI:** 10.3390/life16050740

**Published:** 2026-04-29

**Authors:** Lambros Athanassiou, Ifigenia Kostoglou-Athanassiou, Georgia Kaiafa, Christos Savopoulos, Yehuda Shoenfeld, Panagiotis Athanassiou

**Affiliations:** 1 Department of Rheumatology, St. Paul’s Hospital, 55134 Thessaloniki, Greece; lambros.ath@gmail.com; 2 Department of Endocrinology, Diabetes and Metabolism, Asclepeion Hospital, Voula, 16673 Athens, Greece; ikostoglouathanassiou@yahoo.gr; 3 First Propaedeutic Department of Internal Medicine, AHEPA University General Hospital, Aristotle University of Thessaloniki, 54636 Thessaloniki, Greece; gdkaiafa@yahoo.gr (G.K.); chrisavopoulos@gmail.com (C.S.); 4 Medical School, Reichman University, Herzliya 4610101, Israel; yehuda.shoenfeld@sheba.health.gov.il

**Keywords:** psoriatic arthritis, treatment, biologic agents, TNF inhibitors, IL-17 inhibitors, IL-23 inhibitors, oral small molecules, JAK inhibitors, TYK2 inhibitors, structural damage

## Abstract

Psoriatic arthritis (PsA) is a systemic autoimmune inflammatory disease affecting both the joints and the skin, with the potential involvement of multiple organ systems. A hallmark feature of PsA is enthesitis—inflammation at the sites where tendons and ligaments insert into bone—which arises from a combination of mechanical stress and immune-mediated inflammation. Another defining characteristic of the disease is the paradoxical coexistence of bone erosion and new bone formation, distinguishing it from other inflammatory arthritides. The therapeutic landscape of PsA has evolved considerably over time. Non-steroidal anti-inflammatory drugs (NSAIDs) remain a cornerstone of symptom management, while conventional synthetic disease-modifying antirheumatic drugs (csDMARDs), such as methotrexate, are widely used to control disease progression. The introduction of biologic agents has revolutionized PsA management, with TNF inhibitors, IL-17 inhibitors, and IL-23 inhibitors demonstrating efficacy across a broad range of clinical manifestations. More recently, targeted synthetic small molecules—including JAK inhibitors and TYK2 inhibitors—have expanded the armamentarium of available therapies. The overarching goals of treatment in PsA include the suppression of the underlying inflammatory process and the prevention of structural joint damage. The impact of each therapeutic option on cutaneous psoriasis is an additional and important consideration that guides individualized treatment options.

## 1. Pathogenesis and Clinical Features of PsA

Psoriatic arthritis (PsA) is a systemic inflammatory autoimmune disease which affects primarily the skeleton and the skin [1,2,3]. The disease affects all organ systems [4]. The characteristic of PsA is the involvement of the enthesis, i.e., the point where the tendon is connected to the bone [5,6]. This involvement is in contradistinction to rheumatoid arthritis (RA), which is characterized by the involvement of the joint synovium [7], where the inflammatory process may be initiated. Skeletal manifestations of PsA include oligoarthritis or polyarthritis, enthesitis, dactylitis and axial involvement [4]. The main characteristic of PsA is the simultaneous presence of bone erosion and bone formation [8,9]. This paradox characterizes the disease. Skin involvement in the form of psoriasis may occur first or simultaneously with joint and skeletal involvement or in some cases after the presentation of joint involvement [10,11]. The disease may develop in about 20% of patients with psoriasis.

The exact cause of the disease still evades discovery, despite the multiple efforts via various methods to define it. Genetic and environmental factors seem to be involved and cause a systemic inflammatory autoimmune disease [12,13,14,15]. The disease was treated in earlier times by the administration of non-steroidal-anti-inflammatory drugs (NSAIDs). However, the advent of biologic agents has transformed the treatment of the disease and aim to ameliorate both systemic inflammation and structural damage. The treatment of PsA has evolved constantly in recent decades, with various agents being used in disease management with the aim of combating both inflammation and structural damage. Extra-articular manifestations such as skin involvement or gut damage may govern therapeutic intervention and long-term management.

In earlier years, as well as in very early disease with minimal damage, the application of NSAIDS was a main choice [16]. NSAIDS were used to combat pain and joint edema. Later, conventional synthetic disease-modifying antirheumatic drugs (csDMARDs) such as methotrexate and cyclosporine were introduced in the treatment of PsA [16,17,18]. However, the introduction of biologic agents revolutionized the treatment landscape of PsA [17,18]. TNF inhibitors, in particular etanercept and adalimumab, were applied with success in the treatment of PsA, combating both inflammation and preventing structural damage [19]. Later, as insight into the pathogenesis of PsA developed further, IL-17 inhibitors were introduced in the treatment of PsA [19]. IL-23 inhibitors were also applied [20]. Later, oral small molecules, in particular JAK inhibitors and TYK inhibitors, entered the therapeutic field of PsA treatment [21]. The therapeutic landscape is evolving rapidly with the aim of inhibiting inflammation and preventing structural damage [22]. The presence of skin involvement and the extent of skin lesions in the form of psoriatic lesions is a guide in therapeutic selection. The presence and extent of gut involvement is also a guide in the selection of agents for the therapeutic management of PsA.

## 2. NSAIDS in PsA

NSAIDS are applied in the treatment of PsA for symptom control and the duration of indication is up to 3 months. NSAIDS are an initial choice and are listed in all major guidelines for the treatment of PsA [19]. Agents which may be used are ibuprofen, naproxen, diclofenac, celecoxib and indomethacin. COX-2 inhibitors such as celecoxib have the advantage of a minor risk of gastrointestinal disturbance [23,24], but major cardiovascular risk [25,26]. NSAIDS are not indicated for the inhibition of structural damage or radiographic progression. They may be followed by gastrointestinal disturbance and some of them may be associated with cardiovascular risk. Additionally, psoriasis lesions may progress.

## 3. Conventional Synthetic DMARDS in PsA

Conventional synthetic csDMARDS may be applied as a first-line treatment for PsA. Methotrexate, leflunomide, sulfasalazine and in some cases hydroxychloroquine are used. Methotrexate is considered as a first choice in the treatment of PsA, after the application of NSAIDS [17,18]. However, evidence showing the efficacy of methotrexate, either orally or subcutaneously administered, is not strong.

## 4. Biologic Agents in PsA

Biologic agents have drastically transformed the therapeutic landscape of PsA [18]. They inhibit inflammation and prevent structural damage and radiological progression. TNF inhibitors, IL-17 inhibitors and IL-23 inhibitors have been applied in the therapeutic management of PsA. Treatment choice depends on the clinical findings of PsA and on the simultaneous presence of skin lesions in the form of psoriasis or intestinal involvement.

### 4.1. TNF Inhibitors

TNF inhibitors were the first biologic agents to enter the therapeutic landscape of autoimmune rheumatic diseases, and they were initially applied in rheumatoid arthritis (RA) patients [24]. They were found to inhibit inflammation, disease progression and manage pain, and they were thereafter applied in other autoimmune rheumatic diseases such as spondyloarthritis and PsA [17]. TNF inhibitors suppress inflammation and they are effective for enthesitis, joint and skin involvement. Infliximab, etanercept, adalimumab, certolizumab and golimumab were applied in the treatment of PsA and appeared to be extremely effective in the inhibition of inflammation and in halting structural damage and radiographic progression (Table 1). Pain was also adequately managed by TNF inhibitors.

Infliximab was shown to be effective in the treatment of PsA, having therapeutic efficacy in psoriasis lesions as well [27]. In a trial, the effect of therapeutic infliximab [28], with the dose either standard or based on drug monitoring in patients with autoimmune rheumatic diseases, including 42 patients with PsA, a slightly better therapeutic outcome with the standard dose was observed, although the difference did not reach statistical significance.

Etanercept was applied successfully in the treatment of PsA [29,30,31]. Etanercept was found to be effective in both psoriatic lesions and PsA and to inhibit radiographic progression in PsA without any major safety concerns [32]. Etanercept was found to prevent the progression of psoriasis to PsA [31,33]. Infliximab was used in the treatment of PsA with efficacy [34,35,36]. Infliximab also had beneficial effects on psoriasis [37]. Adalimumab was also applied with success in the treatment of PsA [27,34,35,38,39] and had a beneficial effect on psoriasis [32]. Adalimumab has been shown to modulate T_reg_ function [40]. Golimumab was also used with efficacy in PsA [41]. Certolizumab pegol was also applied with efficacy in patients with PsA and plague psoriasis [42,43].

**Table 1:** Key clinical trials (CT) of TNF inhibitors in PsA.

TNF Inhibitor	Name of CT	Number of Participants	Outcome ACR20	Outcome PASI	Results Radiographic Inhibition
Etanercept	IMPACT	60	73%	significant	yes
Etanercept	IMPACT 2	205	59%	23%	yes
Infliximab	IMPACT	104	65%	significant	yes
Infliximab	IMPACT 2	200	58%	64%	yes
Adalimumab	ADEPT	313	58%	59%	yes
Golimumab	GO-REVEAL	405	51%	56%	yes
Certolizumab	RAPID-PSA	409	58%	significant	yes

ACR20 = American College of Rheumatology 20% response criterion, PASI = Psoriasis Area Severity Index, etanercept [44,45], infliximab [46,47], adalimumab [48,49], golimumab [50,51], certolizumab [52].

### 4.2. Interleukin-23 and Interleukin-17 Inhibitors

As research in the pathophysiology of PsA progressed, it became evident that interleukin-23 and interleukin-17 are critically involved in the pathophysiology of PsA [53,54,55,56,57,58,59]. The inflammatory process is initiated if cells of the innate immune system, in particular dendritic cells and macrophages, respond to microbial products or other signals and secrete interleukin-23 (IL-23). IL-23 is the driver that is involved in the differentiation and further survival of Th 17 cells. IL-23 is also involved in the activation of innate lymphoid cells such as ILC3s and γδ T cells, which are sources of IL-17. The IL-23/IL-17 axis blockade is the basis of PsA treatment [60]. Blockade of either IL-23 or IL-17 is effective, although they target different aspects of the immune cascade [55]. IL-23 inhibitors such as guselkumab and risankizumab act on the adaptive arm of the immune response while IL-17 inhibitors act at the effector arm of the immune response. As already discussed, the hallmark of PsA is enthesitis [59]. Cells of the innate immune system at the enthesis, stimulated mainly by mechanical stress, produce IL-17, a potent inflammatory cytokine.

Secukinumab is an IL-17 inhibitor that has been approved for the treatment of PsA [61]. The FUTURE trial provided evidence for its efficacy on all domains of PsA. The administration of secukinumab induced a significant and sustained reduction in symptoms of PsA, inhibited radiographic progression and improved outcome [61,62,63,64]. Rapid, significant and sustained improvement in PsA manifestations was demonstrated after the administration of secukinumab in biologic-naïve patients and those having had prior TNF inhibitor treatment. Secukinumab was found to be equally effective to adalimumab in musculoskeletal manifestations while it was superior to it on skin manifestations of PsA [65].

Ixekizumab, an IL-17A inhibitor, was approved for PsA in 2017 [66]. Approval was based on the SPIRIT trials which included both biologic-naive and TNF inhibitor inadequate responders [67,68]. Ixekizumab was found to be effective in psoriasis lesions, on peripheral joint symptomatology, and dactylitis, and inhibited structural damage and radiographic progression lesions; however, it did not have the same efficacy on enthesitis [68]. A greater efficacy compared to adalimumab was noted in a head-to-head trial on the simultaneous improvement of joint and skin manifestations [65].

Bimekizumab is a comparatively recently approved agent, which inhibits both IL-17A and IL-17F [69]. It was shown to be effective in the management of PsA and psoriasis [69,70]. In a head-to-head trial in psoriasis patients, it was found to be superior to secukinumab [71]. In indirect comparisons, bimekizumab was predicted to be highly effective in PsA, while in an adjusted indirect comparison in patients naïve to biologics it was found to be more effective than secukinumab [72]. In patients considered inadequate responders to TNF inhibitor, treatment bimekizumab outperformed all agents including guselkumab [73].

Brodalumab, an inhibitor of IL-17RA, blocks IL-17 at the receptor level, which leads to the simultaneous blockade of IL-17A, IL-17E and IL-17F [74]. It is approved for plaque psoriasis and has been shown to be effective in PsA [74,75]. Sonelokimab is a novel agent inhibiting both IL-17A and IL-17F, a nanobody, single-domain antibody fragment, which has been shown to be effective across all domains in PsA [76].

IL-17 inhibitors, including secukinumab, ixekizumab and bimekizumab, reduce joint symptoms, including dactylitis and enthesitis, with bimekizumab demonstrating higher efficacy. IL-17 inhibitors exhibit higher efficacy in psoriasis lesions than TNF inhibitors, with bimekizumab, which is characterized by the dual blockade of IL-17A and IL-17F, exhibiting high efficacy [76]. As far as safety is concerned, dual IL-17A/IL-17F blockade may be associated with oral candidiasis [71]. IL-17A inhibitors should not be considered in patients with active inflammatory bowel or Crohn’s disease as they may aggravate bowel involvement [77].

Based on good efficacy across various domains IL-17, inhibitors may be applied as first-line biologic options for PsA in line with TNF inhibitors, although they may be superior in some domains to them. EULAR 2023 guidelines recommend IL-17 inhibitors as a first-choice biologic DMARD, especially if skin disease or axial involvement predominate [78].

IL-23 is a cytokine composed of two subunitis, P19 and P40, the latter of the two shared with IL-12. It is an inflammatory cytokine with its effects implemented via the JAK-STAT pathway. It promotes the production of cytokines such as IL-17A, IL-17F and IL-22 [79]. Selective IL-23p19 inhibitors, namely guselkumab, risankizumab and tildrakizumab, block the p19 subunit, which is unique to IL-23 and spare IL-12. This characteristic preserves Th1 immune surveillance, which is important for immunity against viruses and tumors and specifically attacks the Th17 axis, which is involved in the pathophysiology of inflammation in PsA. By contrast, ustekinumab, the original agent approved for PsA, binds the p40 subunit and blocks both IL-12 and IL-23.

Ustekinumab is the first IL-23 inhibitor approved for the treatment of PsA in 2013. PSUMMIT trials demonstrated efficacy against PsA [54]. However, the partial blockade of IL-12 and the relatively weak joint response led to it being succeeded by selective p19 agents [19,80].

Guselkumab, a selective IL-23p19 inhibitor, was approved for the treatment of psoriasis in 2017 and for the treatment of PsA in 2020 [81]. Guselkumab was approved after the DISCOVER-1 and DISCOVER-2 trials, which enrolled patients naïve to biologics and patients exposed to TNF inhibitor treatment and patients naïve to biologics with a higher disease burden. Guselkumab exhibited sustained efficacy, with the resolution of dactylitis and enthesitis. Minimal disease activity was also achieved in some patients [80,82] and sustained radiographic protection was also noted. Dactylitis resolution was noted, serum levels of IL-23 were reduced, and it was shown to lead to sustained remission even after treatment withdrawal [83]. Additionally, guselkumab is characterized by dosing flexibility.

Risankizumab was approved by FDA for PsA in 2022. It was approved by the application of the KEEPsAKE program, which established efficacy in patients with a higher treatment failure history [84,85]. Risankizumab has been shown to be effective and safe, with the absence of new safety signals in a trial with a long duration [86]. Risankizumab has a convenient dosing schedule in PsA, with 150 mg sc every 12 weeks after an induction period at weeks 0, 4 and 16, offering a practical advantage.

Tildrakizumab has been approved for plaque psoriasis, has shown efficacy in PsA in a trial and has been approved for PsA in some geographic areas.

Guselkumab and risankizumab have efficacy across both skin and joint domains, with risankizumab showing the greatest skin responses. Guselkumab improved disease activity scores in PsA patients with a history of inadequate responses to TNF inhibitors. IL-17 inhibitors are associated with a faster skin clearance, while IL-23 inhibitors are associated with a long-term durable response. Guselkumab and risankizumab have a robust effect on dactylitis and enthesitis, while the effect of IL-23 inhibitors on axial PsA is currently being investigated [79,87].

The safety profile of IL-23p19 inhibitors is a major advantage, as the safety concerns related to the administration of IL-17 inhibitors, namely oral candidiasis, the induction or worsening of inflammatory bowel disease and suicidal ideation occur infrequently with IL-23p19 inhibitors. Additionally, these IL-23p19 inhibitors have not been associated with an increased risk of tuberculosis reactivation or the triggering of demyelinating disorders, as has been reported with the administration of TNF inhibitors [20]. Selective IL-23 inhibitors are administered with less frequent dosing than IL-17 inhibitors [88]. Adverse events related to IL-23p19 inhibitors are nasopharyngitis and upper respiratory tract infections [20]. In the safety analysis of the DISCOVER trials cases of uveitis, active tuberculosis, infection and inflammatory bowel disease were not observed. The rate of malignancy and major adverse cardiovascular events were not significantly increased [89].

**Figure 1:**
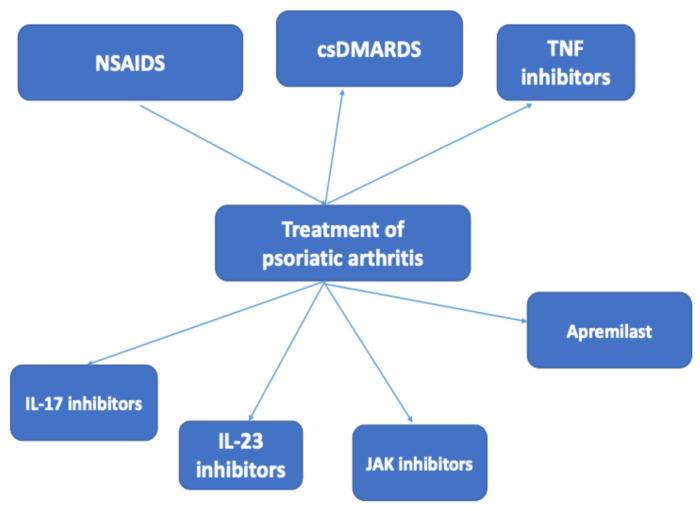
Therapeutic agents used in the treatment of psoriatic arthritis. NSAIDs = non-steroidal-anti-inflammatory drugs, csDMARDs = conventional synthetic disease modifying antirheumatic drugs, TNF = tumor necrosis factor, IL-17 = interleukin-17, IL-23 = interleukin-23.

The availability of various classes of biologic agents for treatment initiation in PsA patients has become a theme for debate (Figure 1) (Table 2). On practical grounds, the presence of inflammatory bowel disease or Crohn’s disease is in favor of the administration of IL-23 inhibitors. The preference of the patient for convenient infrequent dosage administration is in favor of IL-23 inhibitors, particularly risankizumab. In the presence of metabolic syndrome, cardiovascular comorbidity or candidiasis, IL-23 inhibitor treatment may be preferable. In the presence of a high skin disease burden and if skin clearance is a priority, IL-23 inhibitor treatment may be preferable. The presence of axial disease, the need for faster joint relief or a history of failure of IL-23 inhibitor treatment is in favor of IL-17 inhibition; for IL-17, by acting downstream in the immune response, its inhibition may be effective even after IL-23 failure. Anti-IL-17 and anti-IL-23 inhibitors may be preferable over other biologic DMARDs in patients with severe psoriasis, while for arthritis, enthesitis and dactylitis, all biologic agents are applicable [90]. The concept of a sequential strategy is currently being discussed. It may be preferable to initiate treatment with an IL-23 inhibitor, as it is safe and is associated with the possibility of treatment-free remission on some occasions, and to hold as a reserve IL-17 inhibitors as they remain effective after IL-23 inhibitor failure [91].

## 5. JAK Inhibitors in PsA

JAK is the enzyme Janus Kinase, which is located inside the cell and acts as a molecular switch for inflammation [21,100,101,102,103,104]. In the event of a threat detected by the immune system, signaling proteins are released, namely cytokines such as IL-6, IL-12, IL-23 and interferons [104]. These cytokines bind to receptors on the cell surface of immune cells. The effector molecules for these cytokines within the cell are JAK enzymes [102,105]. They are located inside the cell membrane attached to cytokine receptors. In the case of a cytokine attaching on its receptor, the respective JAK enzyme is activated and transmits the signal in the cell via proteins known as STATs (Signal Transducers and Activators of Transcription), which enter the cell nucleus and switch on genes which induce the inflammatory response, in a pathway termed the JAK-STAT signaling cascade [101]. The JAK inhibitor is a small-molecule drug which blocks the JAK enzyme, which results in an interruption of the signaling chain, in effect blocking the inflammatory signal and the inflammatory response [100]. JAK inhibitors are small molecules, in contradistinction to biologic agents, which are large proteins [106,107,108]. Therefore, they may be taken orally, they have quick action and enter the cells directly. There are four members of the JAK family, namely JAK1, JAK2, JAK3 and TYK2 [102]. PsA cytokines like IL-23 and IL-12, signaled via TYK2 and IL-6 via JAK1, are major orchestrators of joint and skin inflammation [109]. By blocking JAK-STAT signaling, JAK inhibitors suppress multiple inflammatory pathways simultaneously with a single oral tablet [108]. Tofacitinib, a JAK1/JAK3 inhibitor, was approved by the FDA in 2017 and by the EMA in 2018 for therapeutic application in patients with PsA [110,111,112,113,114]. It was the first small molecule to receive such an indication. The efficacy of tofacitinib on PsA was confirmed by two trials, namely OPAL Broaden and OPAL Beyond [111]. Tofacitinib was found to be effective across various domains in PsA such as peripheral arthritis, enthesitis, dactylitis and skin manifestations [111]. Additionally, it was found to be effective even in patients who failed on treatment with a biologic agent. JAK inhibitors effectively suppress T cell activation.

Upadacitinib, a selective JAK1 inhibitor, was approved for PsA and represents a significant advance in selectivity as it preferentially inhibits JAK1 [115]. The efficacy of upadacitinib was tested in a significant SELECT-PsA 1 trial which compared upadacitinib head to head with adalimumab in patients naïve to biologics [116]. This head-to-head comparison indicated that upadacitinib was non-inferior and in selected domains superior to adalimumab, in particular skin and enthesitis. The efficacy of upadacitinib was confirmed in biologic non-responders in the SELECT-PsA 2 trial [117].

Deucravacitinib is a selective allosteric TYK2 inhibitor [118]. It binds to the regulatory pseudokinase domain of TYK2. This contrasts with JAK-1/2/3 inhibitors which bind to active kinase sites. This allosteric mechanism confers greater selectivity and avoids JAK2 inhibition, thus leading to a hematologically safer profile. Deucravacitinib is currently approved by the FDA for the treatment of plaque psoriasis. However, it has data demonstrating efficacy in PsA. In the POETYK-PsA Phase 3 trial, deucravacitinib showed efficacy in PsA patients with no new safety signals [119,120]. Only a few hematologic disturbances were observed in the deucravacitinib-treated population.

Brepocitinib is a dual TYK2/JAK1 inhibitor that is administered orally and is effective in patients with plaque psoriasis [121]. It is also administered in the setting of a clinical trial to PsA patients with good results without major adverse events [122]. The JAK1 inhibitors filgotinib and ivarmacitinib are currently in various stages of clinical development for patients with PsA.

Safety is the most critical issue related to the application of JAK inhibitors in PsA [123,124]. Herpes zoster incidence is increased in patients treated with JAK inhibitors. Vaccination against herpes zoster is strongly recommended prior to the initiation of any JAK inhibitor [125]. Major cardiovascular events and venous thromboembolism led to the restriction of pan-JAK inhibitors in patients over 65, those with a cardiovascular risk profile and smokers [22,125]. The risk of malignancy is elevated in high-risk patients, but it is a subject of debate in lower risk patients with PsA [22]. TYK2 inhibitors offer improved selectivity with possibly fewer adverse effects. The hematological and cardiovascular safety profile of deucravacitinib appears favorable.

JAK inhibitors and TYK2 inhibitors hold a place in PsA treatment algorithms [19,126]. They are positioned after conventional synthetic DMARD failure and alongside biologics for moderate to severe disease (Table 3). They are particularly valuable for patients who express preference for oral treatment as compared to injectable biologic agents. They are effective across multiple PsA domains, such as peripheral arthritis, skin, enthesitis, dactylitis and axial disease, being thus suitable for a complex multiple domain disease [19,127]. They are associated with a rapid onset of action.

## 6. Apremilast in PsA

Apremilast, a PDE4 inhibitor, is characterized by anti-inflammatory action [130]. PDE4 is the major enzyme responsible for breaking down cyclic AMP (cAMP), an intracellular second messenger that controls a network of pro- and anti-inflammatory mediators. By inhibiting PDE4, apremilast raises intracellular cAMP levels in immune and non-immune cells, modulating a broad array of inflammatory signals—including reducing TNF-α and IL-23 expression while increasing IL-10 [130]. Apremilast has been approved by the FDA for moderately to severely active psoriatic arthritis and plaque psoriasis [131,132]. The efficacy of apremilast in PsA has been shown in the PALACE trials [133]. It is a relatively safe therapeutic agent whose main adverse effects are diarrhea and nausea [131]. Neuropsychiatric effects such as depression may develop and should be considered and monitored. Its efficacy is lower than that of biologic treatments; however, due to the favorable safety profile, it may be applied in patients at high risk of infection or a contraindication to biologics [134].

## 7. Cellular Treatment in PsA

Recent research has highlighted the importance of regulatory T cells, known as Tregs, in immune regulation [135,136]. It appears that tissue-residing Tregs play an essential role in the integration of the immune response and may maintain organ homeostasis via immune and metabolic signals. Therapeutic advances, such as chimeric antigen receptor CAR/T cells, have been shown to inhibit severe autoimmune inflammatory diseases such as systemic lupus erythematosus [137] and are investigated for their possible role in the treatment of severe PsA [138].

## 8. Complementary, Alternative Therapies in PsA

Complementary or alternative modes of treatment are applied in PsA. The effect of omega-3 fatty acids on inflammation has been extensively investigated [139,140,141]. It was observed that they may have mild anti-inflammatory properties and are thought to have no major adverse effects. Vitamin D has been shown to have anti-inflammatory properties and to exert preventive as well as therapeutic effects on systemic autoimmune inflammatory diseases [142,143]. Alterations in vitamin D levels and metabolism have been noted in patients with PsA and it is thought that vitamin D metabolism should be evaluated further in PsA [144]. Low-dose naltrexone is known to modulate cytokine levels and lymphocyte function and may be applied as an alternative mode of treatment in psoriatic disease [145,146].

## 9. Lifestyle Modification

PsA is frequently accompanied by metabolic syndrome and obesity, which aggravate its clinical picture [147,148,149]. Obesity may induce systemic inflammation and may aggravate pain [150]. Therefore, various measures should be applied to prevent or treat obesity and metabolic syndrome. Exercise and physical therapy should be implemented to preserve articular function. The administration of GLP-1 receptor agonists for the treatment of obesity may contribute to the management of metabolic syndrome and the underlying systemic inflammation [151].

## 10. Discussion

PsA is a systemic autoimmune inflammatory disease with articular and cutaneous manifestations, which may develop in the context of pre-existing psoriasis [10]. It is a multidomain condition characterized by considerable phenotypic heterogeneity, with the potential involvement of the axial skeleton, peripheral joints, skin, and other organ systems [4]. The disease imposes a significant burden of pain and substantially impairs quality of life [1]. Over recent decades, therapeutic advances have been rapid and continue to evolve, in parallel with a deepening understanding of disease pathophysiology and the identification of novel molecular targets [15] (Table 4).

In earlier years, management relied primarily on NSAIDs and conventional synthetic DMARDs [16]. The introduction of TNF inhibitors in the treatment of autoimmune rheumatic diseases marked a pivotal advance, with their subsequent successful application in PsA [18]. Beyond their anti-inflammatory effects, TNF inhibitors favorably modulate bone metabolism by concurrently suppressing pathological new bone formation and bone resorption [39,152,153]. Apremilast has further enriched the therapeutic armamentarium through its inhibition of inflammatory osteoclastogenesis [154].

Elucidation of the critical role of IL-23 in the pathophysiology of the disease led to the discovery of various IL-23 inhibitors and IL-17 inhibitors in the treatment of PsA [11,14]. Research on the JAK-STAT signaling pathway led to the successful application of JAK inhibitors in the treatment of PsA [127]. Further research is necessary for the elucidation of the factors leading to the progression of psoriasis to PsA and the therapeutic agents which may prevent this progression [113]. The current therapeutic landscape, TNF inhibitors, IL-17 inhibitors, IL-23 inhibitors and JAK inhibitors block bone erosion, but their effect in new bone formation is debatable and currently under investigation. Novel agents which block bone erosion and simultaneously target new bone formation are under evaluation.

Therapeutic selection in PsA is guided by a comprehensive assessment of disease domains, including the presence and extent of peripheral joint involvement, axial disease, cutaneous lesions, and concomitant gut inflammation [91]. Patient preferences and the feasibility of oral administration also inform treatment decisions [104]; in this regard, agents such as apremilast and JAK inhibitors offer effective oral alternatives [19]. Furthermore, certain agents with convenient dosing schedules provide additional flexibility and may be particularly suited to specific patient populations [1].

## 11. Conclusions

PsA, a systemic progressive autoimmune inflammatory disease which affects the joints, the axial skeleton and the skin, is currently treated multimodally. NSAIDs and conventional synthetic DMARDs are used in disease management. Biologic DMARDs, namely TNF inhibitors, are applied successfully for the management of joint and skin symptoms and for the inhibition of structural damage as well as radiological progression. IL-23p19 inhibitors are also applied in the treatment of PsA. IL-17 inhibitors have also a prominent place in the treatment of PsA. JAK inhibitors, including TYK2 inhibitors, are administered orally and are a convenient and effective treatment modality. The disease is a multidomain disease and may have a varying clinical expression. The presence of skin involvement and gut involvement should be taken into account, and it should guide therapeutic selection. Despite the recent therapeutic developments, the varying clinical phenotype and multidomain nature of the disease necessitate continuous research efforts to treat it effectively.

## Figures and Tables

**Table 2:** Key clinical trials of IL-23 and IL-17 inhibitors in PsA.

Clinical Trial	N	Outcome	Results
IL-23 inhibitor Guselkumab DISCOVER-1	381	ACR20 at 24 weeks	ACR20: 59% (q4w) · 52% (q8w) vs. 22% placebo. Included TNFi-experienced patients; similar response regardless of prior TNFi use. No discontinuations due to lack of efficacy.
IL-23 inhibitor Guselkumab DISCOVER-2	739	ACR20 at 24 weeks	ACR20: 64% (q4w) · 64% (q8w) vs. 33% placebo (*p* < 0.0001). Biologic-naïve only. Durable efficacy confirmed at 100 weeks across joint, skin, and entheseal domains.
IL-23 inhibitor Risankizumab KEEPsAKE-1	964	ACR20 at 24 weeks	ACR20: 57.3% vs. 33.5% placebo Biologic-naïve patients. Significant improvements in dactylitis, enthesitis, and PASI scores.
IL-23 inhibitor Risankizumab KEEPsAKE-2	444	ACR20 at 24 weeks	ACR20: 51.3% vs. 26.5% placebo Prior inadequate response to ≥1 biologic (TNFi or IL-12/23i). Consistent benefit in biologic-experienced population.
IL-12/23 inhibitor Ustekinumab PSUMMIT-1	615	ACR20 at 24 weeks	ACR20: 42% (45 mg) · 50% (90 mg) vs. 23% placebo Biologic-naïve. Established IL-12/23 blockade in PsA; significant skin and joint improvement.
IL-12/23 inhibitor Ustekinumab PSUMMIT-2	312	ACR20 at 24 weeks	ACR20: 44% (45 mg) · 44% (90 mg) vs. 20% placebo TNFi-experienced.
IL-17A inhibitor Secukinumab FUTURE-1	606	ACR20 at 24 weeks	ACR20: 50% (IV → 150 mg) · 50% (IV → 75 mg) vs. 17% placebo. Rapid onset of response. Benefits sustained at week 52 in both doses.
IL-17A inhibitor Secukinumab FUTURE-2	397	ACR20 at 24 weeks	ACR20: 54% (300 mg) · 51% (150 mg) · 29% (75 mg) vs. 15% placebo. Dose-dependent response; 300 mg dose showed superior skin clearance. TNFi-naïve and experienced included.
IL-17A inhibitor Ixekizumab SPIRIT-P1	417	ACR20 at 24 weeks	ACR20: 62% (q2w) · 58% (q4w) vs. 30% placebo. Biologic-naïve. Significant improvements in enthesitis, dactylitis, and skin.
IL-17A inhibitor Ixekizumab SPIRIT-P2	363	ACR20 at 24 weeks	ACR20: 53% (q2w) · 48% (q4w) vs. 20% placebo TNFi-experienced patients. Consistent efficacy in refractory disease.
IL-17A/RA inhibitor Brodalumab AMAGINE-1/2/3	~1.400	ACR20 at week 16	ACR20: ~55–60% (210 mg q2w) vs. ~27% placebo Blocks IL-17RA receptor. Superior skin clearance.
Dual IL-17A/F inhibitor Bimekizumab BE OPTIMAL	852	ACR50 at week 16	ACR50: 43.9% vs. 10.0% placebo (*p* < 0.0001) Biologic-naïve. Dual IL-17A/F blockade showed enhanced ACR70 vs. secukinumab in indirect comparison (OR 2.39).
Dual IL-17A/F inhibitor Bimekizumab BE COMPLETE	400	ACR50 at week 16	ACR50: 43.4% vs. 6.8% placebo (*p* < 0.0001) TNFi-experienced.

N = Number of participants, ACR20 = American College of Rheumatology 20% response criterion, guselkumab [80,92], Risankizumab [84,85], ustekinumab [93], secukinumab [94,95], ixekizumab [96,97], brodalumab [98], bimekizumab [99].

**Table 3:** Clinical trials of JAK-STAT inhibitors in psoriatic arthritis.

Clinical Trial	N	Outcome	Results
AK1/3 inhibitor Tofacitinib OPAL Broaden	422	ACR20 + HAQ-DI at month 3	ACR20: 50% (5 mg) · 61% (10 mg) vs. 33% placebo; adalimumab comparator arm: 52%. csDMARD-IR (biologic-naïve). Both doses are noninferior to adalimumab. 91–98% of patients showed no radiographic progression at 12 months.
AK1/3 inhibitor Tofacitinib OPAL Beyond	395	ACR20 + HAQ-DI at month 3	ACR20: 50% (5 mg) · 47% (10 mg) vs. 24% placebo (*p* < 0.001) TNFi-experienced (biologic-refractory). PASI75 superior only with 10 mg (43% vs. 14%).
JAK1 inhibitor Upadacitinib SELECT-PsA 1	1704	ACR20 at week 12	ACR20: 70.6% (15 mg) · 78.5% (30 mg) vs. 36.2% placebo; adalimumab arm: 65% csDMARD-IR (biologic-naïve). 30 mg was superior to adalimumab; 15 mg was noninferior.
JAK1 inhibitor Upadacitinib SELECT-PsA 2	642	ACR20 at week 12	ACR20: 56.9% (15 mg) · 63.8% (30 mg) vs. 24.1% placebo (*p* < 0.001) Biologic-refractory (TNFi-experienced). MDA at week 24: 25.1% (15 mg) and 28.9% (30 mg) vs. 2.8% placebo. Consistent efficacy across biologic-experienced population.
TYK2 inhibitor Deucravacitinib FDA approved Mar 2026 POETYK PsA-1	670	ACR20 at week 16	ACR20: 54.2% vs. 34.1% placebo (*p* < 0.0001); PASI75: 51.9% vs. 7.1%. bDMARD-naïve. Responses deepened through week 24 and were maintained at week 52 (63.1%). Structural damage inhibition confirmed.
TYK2 inhibitor Deucravacitinib FDA approved Mar 2026 POETYK PsA-2	~730	ACR20 at week 16	ACR20: 54.2% vs. 39.4% placebo (*p* = 0.0002); ACR20 at week 52: 62.2% (continuous) · 67.3% (switched from placebo). bDMARD-naïve or TNFi-experienced.

N = number of participants, ACR20 = American College of Rheumatology 20%. response criterion. Tofacitinib [110,114], upadacitinib [117,128], deucravacitinib [129].

**Table 4:** Targets of the therapeutic agents applied in PsA.

Therapeutic Agents	Target
TNF inhibitors	TNF-α
IL-23 inhibitors	IL-23
IL-17 inhibitors	IL-17
JAK-STAT inhibitors	JAK enzyme
apremilast	PDE4 enzyme

## Data Availability

No new data were created or analyzed in this study. Data sharing is not applicable to this article.
